# A deterministic detector for vector vortex states

**DOI:** 10.1038/s41598-017-12739-z

**Published:** 2017-10-24

**Authors:** Bienvenu Ndagano, Isaac Nape, Benjamin Perez-Garcia, Stirling Scholes, Raul I. Hernandez-Aranda, Thomas Konrad, Martin P. J. Lavery, Andrew Forbes

**Affiliations:** 1 School of Physics, University of the Witwatersrand, Private Bag 3, Wits, 2050 South Africa; 2 Photonics and Mathematical Optics Group, Tecnologico de Monterrey, Monterrey, 64849 Mexico; 3 College of Chemistry and Physics, University of KwaZulu-Natal, Private Bag X54001, Durban, 4000 South Africa; 4 School of Engineering, University of Glasgow, Glasgow, G12 8QQ Scotland United Kingdom

## Abstract

Encoding information in high-dimensional degrees of freedom of photons has led to new avenues in various quantum protocols such as communication and information processing. Yet to fully benefit from the increase in dimension requires a deterministic detection system, e.g., to reduce dimension dependent photon loss in quantum key distribution. Recently, there has been a growing interest in using vector vortex modes, spatial modes of light with entangled degrees of freedom, as a basis for encoding information. However, there is at present no method to detect these non-separable states in a deterministic manner, negating the benefit of the larger state space. Here we present a method to deterministically detect single photon states in a four dimensional space spanned by vector vortex modes with entangled polarisation and orbital angular momentum degrees of freedom. We demonstrate our detection system with vector vortex modes from the |${\boldsymbol{\ell }}$| = 1 and |${\boldsymbol{\ell }}$| = 10 subspaces using classical and weak coherent states and find excellent detection fidelities for both pure and superposition vector states. This work opens the possibility to increase the dimensionality of the state-space used for encoding information while maintaining deterministic detection and will be invaluable for long distance classical and quantum communication.

## Introduction

Employing spatial modes to push higher the ceiling of achievable bandwidth has shown considerable improvements in classical communication systems; for example with light carrying orbital angular momentum (OAM)^1–8^. At the quantum level, the high dimensional state space of spatial modes carry the promise of high capacity communication with single photons. Quantum key distribution (QKD) is one of the many vibrant fields that would greatly benefit from the increase in dimension^9,10^. Going beyond the realm of OAM modes, photons with exotic spatial and polarisation structure, commonly known as vector modes, have been used as information carriers for polarisation encoded qubits in alignment-free QKD^11–13^. Taking advantage of the rotational symmetry of OAM vector modes, it was shown that the transverse axes of detectors need not to be aligned in order to reconcile the encoding and decoding bases, as would be the case in QKD with only polarisation. In these vector modes, the spatial and polarisation degrees of freedom (DoFs) are coupled in a non-separable manner, reminiscent of entanglement in quantum mechanics. This non-separability can be used to encode information and has been done so with classical light^14,15^, for example, in mode division multiplexing^16^.

However, realizing high-dimensional quantum communication remains challenging. To date, the list of reports on high dimensional QKD with spatial modes is not exhaustive, and include protocols in up to *d* = 7^17–20^. One of the important issues to address and which is crucial to QKD, is the efficient detection of the prepared photon. It is well known that scalar spatial modes can be detected probabilistically with holographic filters that can be encoded on a spatial light modulator (SLM)^8^, and deterministically with mode sorters^21^. In contrast, the detection of vector vortex modes is rather more complex, with tools existing at the many photon level^16,22^ but none to date for the deterministic detection at the single photon level. For example, Milione *et al*. have shown that when using *q*-plates to prepare and detect vector vortex modes, one needs two oppositely charged *q*-plates to detect all the degenerate modes, resulting in pairs of incompatible projections and a 50% probability of detection^16^. In the context of QKD, such a probabilistic detection would result in lower sift rates, cancelling the benefits of the increased dimensionality^18^.

Here we introduce a new detection scheme that, deterministically and without dimension dependent photon loss, can detect all basis elements in our high-dimensional vector mode space. We achieve this through a two step process: using polarisation as a marker, we first sort the vector modes in two paths according to their intra-modal phase (0 or *π*) through interference. The OAM carried by the photon in each path is then mapped to position using refractive OAM mode sorters^21,23–25^. The mapping of vector mode to position allows for the deterministic detection of higher dimensional state spaces, a feature particularly beneficial to QKD as it allows for high sifting rates. Likewise with classical communication where an improvement to signal-to-noise ratio over existing detection methods would be expected because the received signal is no longer distributed across many detectors. To demonstrate the detector, we present a simple prepare-and-measure QKD BB84 protocol using vector modes–and their mutually unbiased OAM scalar modes–within the $|\ell |=1$ and $|\ell |=10$ subspaces.

## Results

### High dimensional encoding with vector vortex modes

**Figure 1:**
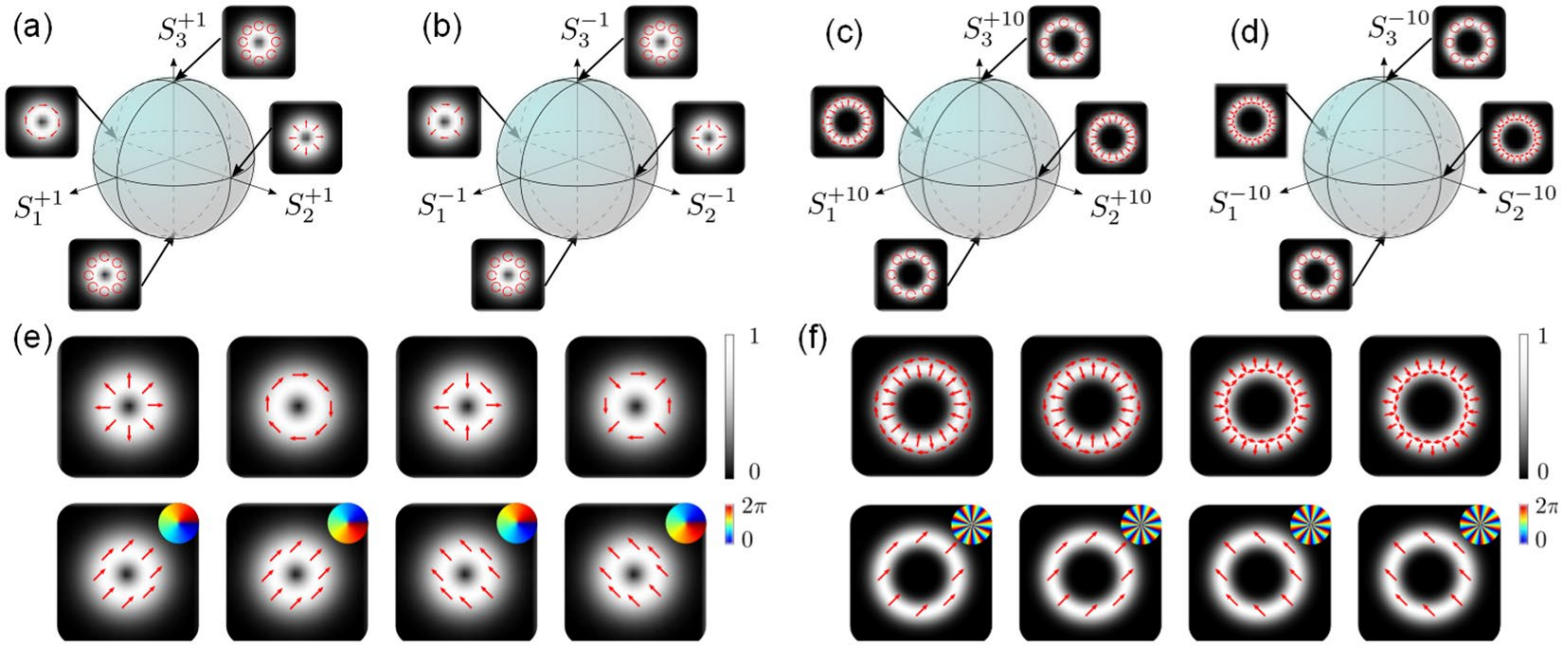
High–order Poincaré sphere (HOPS). We show the HOPS for the cases: (**a**) $\ell =1$, (**b**) $\ell =-1$, (**c**) $\ell =10$ and (**d**) $\ell =-10$. (**e**,**f**) Show mutually unbiased vector and scalar vortex modes from, the $\ell =\pm 1$ and $\ell =\pm 10$ subspaces, respectively. The insets show the azimuthally varying phase profile of the scalar/OAM modes. Observe that the modes corresponding to the maximally entangled local local Bell states occur on the equator of the HOPS.

Early QKD demonstrations were performed using the polarisation DoF, namely, states in the space spanned by left-circular $|L\rangle$ and right-circular polarisation $|R\rangle$, i.e., ${ {\mathcal H} }_{\sigma }=span\,\{|L\rangle ,|R\rangle \}$, and later using the spatial mode of light as a DoF, e.g., OAM with ${ {\mathcal H} }_{\ell }=span\,\{|\ell \rangle ,|-\ell \rangle \}$. Using entangled states in both DoFs allows one to access a large state space, ${ {\mathcal H} }_{\sigma ,\ell }={ {\mathcal H} }_{\sigma }\otimes { {\mathcal H} }_{\ell }$, described by the higher-order Poincaré sphere (HOPS)^26,27^, as shown graphically in Fig. 1(a)–(d). Employing multiple OAM values the final state space is a direct sum of the subspaces ${ {\mathcal H} }_{\sigma ,\ell }$ for different $\ell$:

1 $${ {\mathcal H} }_{M}=\mathop{\oplus }\limits_{\ell \in M}{ {\mathcal H} }_{\sigma ,\ell },$$

where $M\subset {\mathbb{N}}$ and the direct sum ⊕ of vector spaces *A*_*i*_ is given by ${\oplus }_{i}{A}_{i}=\{{\sum }_{i}\,{\alpha }_{i}|{a}_{i}\rangle :|{a}_{i}\rangle \in {A}_{i},{\alpha }_{i}\in {\mathbb{C}}\}$. The dimension of ${ {\mathcal H} }_{M}$ equals the sum of the dimensions of the subspaces ${ {\mathcal H} }_{\sigma ,\ell }$, i.e. *d* = 4*N*, with *N* = |Ω| being the number of different values of $|\ell |$ (the distinct subspaces or spheres). This opens the way to infinite dimensional encoding using such hybrid states. For example, using only the $|\ell |$ subspace of OAM leads to a four dimensional space spanned by $\{|\ell ,\,L\rangle ,\,|-\ell ,\,L\rangle ,\,|\ell ,\,R\rangle ,\,|-\ell ,\,R\rangle \}$. It is precisely in this four-dimensional subspace that, here, we define our vector and scalar modes: a vector mode set, ${|\psi \rangle }_{\ell ,\theta }$, which forms four local local Bell states with entangled internal degrees of freedom, and a mutually unbiased scalar mode set, ${|\varphi \rangle }_{\ell ,\theta }$, defined as

2 $${|\psi \rangle }_{\ell ,\theta }=\frac{1}{\sqrt{2}}(|R\rangle |\ell \rangle +{e}^{i\theta }|L\rangle |-\ell \rangle ),$$

3 $${|\varphi \rangle }_{\ell ,\theta }=\frac{1}{\sqrt{2}}\,(|R\rangle +{e}^{i(\theta -\tfrac{\pi }{2})}|L\rangle )\,|\ell \rangle ,$$

where *θ* = 0 or *π* is the intra-modal phase. For a given $|\ell |$ OAM subspace, there exist a set of four orthogonal modes in both the vector basis (Eq. 2) and its mutually unbiased counterpart (Eq. 3), such that |〈*ψ*|*ϕ*〉|^2^ = 1/*d* with *d* = 4. Both mode sets can be generated by manipulating the dynamic or geometric phase of light^8,28–30^. Here we employ geometric phase control through a combination of *q*-plates^31,32^ and wave plates to create all vector modes in the four dimensional subspace defined by a fixed $|\ell |$. For the purpose of demonstration, we use vector and scalar modes in the $\ell =\pm 1$ and $\ell =\pm 10$ OAM subspaces, shown graphically in Fig. 1(e,f) respectively. Table 1 details the combination of wave-plate and *q*-plates required to generate the vector modes in Fig. 1, given an input, horizontally polarised, Gaussian laser beam. For example, to produce a ${|\psi \rangle }_{-\mathrm{10,}\pi }$ state, the horizontally polarised Gaussian laser beam is passed through HWP1 oriented at *π*/4 radians, then a *q*-plate with *q* = 5 and finally through HWP2 oriented at 0 radian.

**Table 1:** Requirements for the generation of each of the four local local Bell states in the two subspaces of $|\ell |=1$ and $|\ell |=10$.

Bell State	HWP1 angle (*γ*_1_)	HWP2 angle (*γ*_2_)	*q*
${|\psi \rangle }_{\mathrm{1,0}}$	0	—	0.5
${|\psi \rangle }_{\mathrm{1,}\pi }$	*π*/4	—	0.5
${|\psi \rangle }_{-\mathrm{1,0}}$	0	0	0.5
${|\psi \rangle }_{-\mathrm{1,}\pi }$	*π*/4	0	0.5
${|\psi \rangle }_{\mathrm{10,0}}$	0	—	5
${|\psi \rangle }_{\mathrm{10,}\pi }$	*π*/4	—	5
${|\psi \rangle }_{-\mathrm{10,0}}$	0	0	5
${|\psi \rangle }_{-\mathrm{10,}\pi }$	*π*/4	0	5

**Figure 2:**
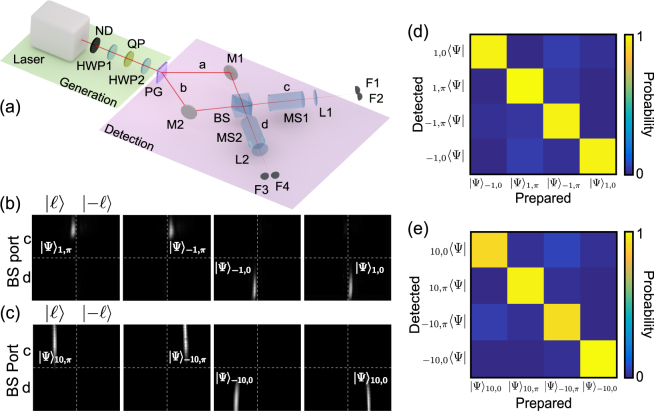
Deterministic detection of vector vortex modes. (**a**) A vector beam is generated by shaping attenuated light from a He-Ne laser with geometric phase optics: half-wave plates (HWP1 and HWP2) and a *q*-plate (QP). A polarisation grating (PG) maps circular polarisation to two paths, *a* and *b*, which are then interfered at a 50:50 beam-splitter (BS). Subsequently the OAM states are measured using mode sorters (MS1 and MS2) that map OAM to position. The output ports (*c* or *d*) of the BS and the lateral locations $(|\ell \rangle \,{\rm{or}}\,|-\ell \rangle )$ deterministically indicate the Bell state being measured by the detectors F1–F4. Experimental results from the spatial sorting of vector modes are shown in (**b**) for $\ell =\pm 1$ and (**c**) for $\ell =\pm 10$, with crosstalk matrices for these subspaces shown in (**d**,**e**). M1 and M2 are mirrors; L1 and L2 are lenses, and F1–F4 are multimode fibers.

The input state is a linearly polarised Gaussian beam and all angles are defined with respect to the horizontal axis. The experimental arrangements of the wave plates and q-plates is shown in Fig. 2(a).

### A deterministic detector for classical and quantum vector modes

We now introduce a new scheme to deterministically detect the vector vortex modes, as detailed in Fig. 2(a), that has a number of practical advantages for classical and quantum communication. Consider a vector mode as defined in Eq. 2. The sorting of the different vector modes is achieved through a combination of geometric phase control and multi-path interference. First, a polarisation grating^33^ based on geometric phase acts as a beam splitter for left- and right-circularly polarised photons, creating two paths (*a* and *b*)

4 $${|\psi \rangle }_{\ell ,\theta }\to \frac{1}{\sqrt{2}}({|R\rangle }_{a}{|\ell \rangle }_{a}+{e}^{i\theta }{|L\rangle }_{b}{|-\ell \rangle }_{b}),$$

where the subscript *a* and *b* refer to the polarisation-marked paths. The photon paths *a* and *b* are interfered at a 50:50 BS, resulting in the following state after the BS:

5 $${|\psi ^{\prime} \rangle }_{\ell ,\theta }=\frac{1+{e}^{i(\delta +\theta +\frac{\pi }{2})}}{2}{|\ell \rangle }_{c}{|R\rangle }_{c}+i\frac{1+{e}^{i(\delta +\theta -\frac{\pi }{2})}}{2}{|-\ell \rangle }_{d}{|L\rangle }_{d}$$

where the subscripts *c* and *d* refer to the output ports of the beam splitter and *δ* is the dynamic phase difference between the two paths. Note that the polarisation of the two paths is automatically reconciled in each of the output ports of the beam splitter due to the difference of parity in the number of reflections for each input arm. Also note that at this point it is not necessary to retain the polarisation kets in the expression of the photon state since the polarisation information is contained in the path. In our setup we set *δ* = *π*/2, reducing the state in Eq. 5 to

6 $${|\psi ^{\prime} \rangle }_{\ell ,\theta }=\frac{1-{e}^{i\theta }}{2}{|\ell \rangle }_{c}+i\frac{1+{e}^{i\theta }}{2}{|-\ell \rangle }_{d}$$

The measurement system is completed by passing each of the outputs in *c* and *d* through a mode sorter to path split the positive and negative $\ell$ states, and collecting these photons using 4 fibres coupled to photodiodes F_*i*_. The mode sorters are refractive (lossless) aspheres that map OAM to position^21,23–25^. The first optical element of the sorter unwraps the OAM modes while the second acts a phase corrector by stopping the unwrapping process. The results is a transformation from an azimuthal to linear phase with a gradient proportional to the OAM charge incident on the sorter. The modes map according to

7 $${|\psi \rangle }_{\ell \mathrm{,0}}\to i{|-\ell \rangle }_{d}\to {{\rm{F}}}_{4},$$

8 $${|\psi \rangle }_{\ell ,\pi }\to {|\ell \rangle }_{c}\to {{\rm{F}}}_{2},$$

9 $${|\psi \rangle }_{-\ell \mathrm{,0}}\to i{|\ell \rangle }_{d}\to {{\rm{F}}}_{3},$$

10 $${|\psi \rangle }_{-\ell ,\pi }\to {|-\ell \rangle }_{c}\to {{\rm{F}}}_{1}.$$

where the indices *c* and *d* label the output ports of the BS. For example, the Bell state ${|\psi \rangle }_{\ell \mathrm{,0}}$, which is known as radially polarised light at the classical level, is directed to path *d* and detector F3 $(|-\ell \rangle )$, thus making the detection deterministic, and likewise for all the other states. This is shown in Fig. 2(b,c) for the two subspaces $\ell =\pm 1$ and $\ell =\pm 10$, where all the vector modes under study are mapped to unique positions at the detector plane. While it is trivial to measure such vector states at the classical level with many photons^14,16,22^, with our approach each single photon Bell state is detected with, in principle, unit probability at the single photon level. Figure 2(d,e) shows the probability of detection of single photons for different HOPS local local Bell states ($\ell =\pm 1$ and $\ell =\pm 10$). The depicted results validates our proposal as a highly efficient method to sort local Bell states with in principle unit probability. Observe that the detected states are in excellent agreement with the generated local Bell states for all cases. In classical optics such non–separable states, also known as vector vortex beams, have been widely studied. Under this regime our detection apparatus also works but with a *d*× signal-to-noise as the incoming signal is not distributed across *d* detectors, where *d* is the number of classical modes in the set.

**Figure 3:**
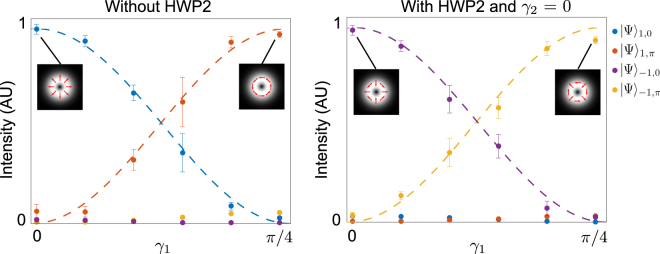
Detection of superpositions of vector states. The two graphs show detection (normalised intensity) of superpositions of states from our vector basis. The superpositions were created by changing the HWP angle *γ*_1_ (cp. Table 1). Each point was generated by averaging 30 measurements. The dashed lines represent the theoretical curve.

Finally, Fig. 3 shows the results for the detection of photons in superpositions of our vector basis. This task is performed by changing the angle *γ*_1_ of the HWP1 and measuring the intensity in each output port of the setup. The experimental results (data points) are in excellent agreement with theory (dashed curves), validating that arbitrary superpositions may also be detected with high fidelity. Note that the detection of the mutually unbiased scalar mode works in an analogous manner, with the exception that the BS is removed since there is no need to resolve intermodal phases to recover the vector nature of the mode.

### Application to quantum key distribution

We performed a four dimensional prepare-and-measure BB84 QKD protocol^34^ using the mutually unbiased vector and scalar modes given earlier, namely, a vector mode set, ${|\psi \rangle }_{\ell ,\theta }$, and a mutually unbiased scalar mode set, ${|\varphi \rangle }_{\ell ,\theta }$. For a given $|\ell |$ OAM subspace, there exists a set of four orthogonal modes in both the vector basis (Eq. 2) and its mutually unbiased counterpart (Eq. 3). Our four vector modes for QKD then become:

11 $${|00\rangle }_{v}=\frac{1}{\sqrt{2}}(|R\rangle \,|\ell \rangle +|L\rangle \,|-\ell \rangle ),$$

12 $${|01\rangle }_{v}=\frac{1}{\sqrt{2}}(|R\rangle \,|\ell \rangle -|L\rangle \,|-\ell \rangle ),$$

13 $${|10\rangle }_{v}=\frac{1}{\sqrt{2}}(|L\rangle \,|\ell \rangle +|R\rangle \,|-\ell \rangle ),$$

14 $${|11\rangle }_{v}=\frac{1}{\sqrt{2}}(|L\rangle \,|\ell \rangle -|R\rangle \,|-\ell \rangle ),$$

with corresponding mutually unbiased bases (MUB)

15 $${|00\rangle }_{s}=|D\rangle \,|-\ell \rangle ,$$

16 $${|01\rangle }_{s}=|D\rangle \,|\ell \rangle ,$$

17 $${|10\rangle }_{s}=|A\rangle \,|-\ell \rangle ,$$

18 $${|11\rangle }_{s}=|A\rangle \,|\ell \rangle ,$$

where the subscripts *s* and *v* refer to, respectively, the scalar and vector mode basis, while *D* and *A* are the diagonal and anti-diagonal polarisation states.

**Figure 4:**
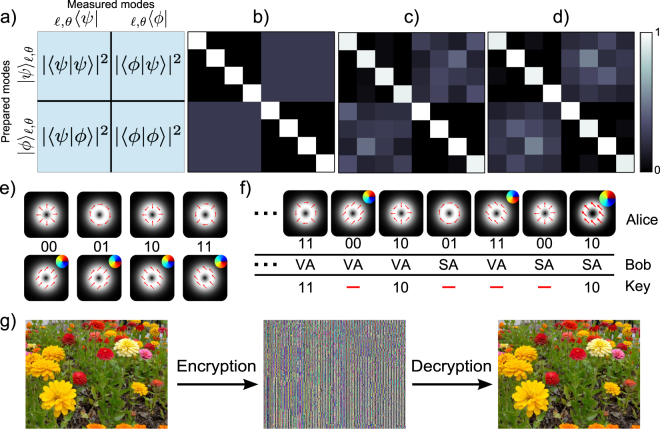
High dimensional QKD with vector modes. (**a**) Schematic of the inner product measurements performed between the vector states and their mutually unbiased counterparts, (**b**) theoretical scattering probabilities among the vector and scalar modes following the measurement procedure in (**a**), and experimental results for the (**c**) $\ell =\pm 1$ and (**d**) $\ell =\pm 10$ subspaces. (**e**) Alice and Bob agree on bit values for the vector and scalar modes. (**f**) Alice sends a random sequence of vector and scalar modes, which Bob randomly measures using either a vector analyser (VA) or a scalar analyser (SA). Alice and Bob, upon communication of the encoding and decoding bases through a classical channel, discard bit values for modes prepared and measured in complementary bases. (**g**) Shows a simple encryption/decryption of an image using a 98 bit long key, sifted from a total of 200 transmitted bits. Photo courtesy photos-public-domain.com ^39^.

Light from our source was attenuated to an average photon number of *μ* = 0.008 per pulse. Note that while such weak coherent states cannot be used for QKD without photon splitting strategies, it is a suitable demonstration of the detection scheme, which is our aim here. Alice prepared an initial state in either the vector or scalar basis and transmitted it to Bob, who made his measurements as detailed in the previous section with deterministic vector and scalar mode detectors. Through optical projection onto both the vector and scalar bases as illustrated in Fig. 4(a), we determined the crosstalk matrices shown theoretically in Fig. 4(b) and experimentally in (c) and (d), relating the input and measured modes within, respectively, the subspaces $\ell =\pm 1$ and $\ell =\pm 10$. The average fidelity of detection, measured for modes prepared and detected in identical bases, is *F* = 0.965 ± 0.004 while the overlap between modes from mutually unbiased bases is |〈*ϕ*|*ψ*〉|^2^ = 0.255 ± 0.004, in good agreement with the theoretical value of 0.25.

For each mode, Alice and Bob assign the bit values 00,01,10 and 11, as shown in Fig. 4(e). During the transmission, Alice randomly prepares her photon and Bob randomly measures the photon with either the vector or scalar analyser (the scalar analyser is the vector analyser discussed earlier but without the beam splitter). At the end of the transmission, Alice and Bob reconcile the prepare and measure bases and discard measurements in complementary bases, as illustrated in Fig. 4(f). We performed this transmission using a sequence of 100 modes and retained a sifted key of 49 spatial modes (98 bits), which was used to encrypt and decrypt a picture as shown in Fig. 4(g).

From the measured crosstalk matrices in Fig. 4(c,d), we performed a security analysis on our QKD scheme in dimension *d* = 4 for the two OAM subspaces (±1 and ±10). The results of the analysis are summarised in Table 2.

**Table 2:** Summary of the security analysis on the high dimensional protocol showing the experimental and theoretical values of the detection fidelity (*F*), mutual information *I*_*AB*_ between Alice and Bob, Eve’s cloning fidelity (*F*_*E*_) and mutual information with Alice *I*_*AE*_, as well as the quantum error rate *Q* and information capacity per photon *R* in bits per photon.

Measures	*d* = 4 (${\boldsymbol{\ell }}{\boldsymbol{=}}{\boldsymbol{\pm }}{\bf{1}}$)	*d* = 4 (${\boldsymbol{\ell }}{\boldsymbol{=}}{\boldsymbol{\pm }}{\bf{10}}$)	ideal
	experiment	experiment	
*F*	0.96	0.97	1.00
*I*_*AB*_	1.69	1.76	2.00
*F*_*E*_	0.44	0.41	0.25
*I*_*AE*_	0.17	0.13	0.00
*Q*	0.04	0.03	0.00
*R*	1.39	1.52	2.00

The lower bound on the information capacity per photon yields a value as high as 1.52 bits per photon. While the security of the protocol can be increased with privacy amplification, the measured four-dimensional secret key rate demonstrates the potential of such non-separable modes for high bandwidth quantum communication.

We emphasise that the above demonstration serves to outline the benefits of this basis set for QKD, which is now feasible given the deterministic detector proposed here. Our work highlights that the deterministic detection of vector vortex modes would result in higher sift rates when compared to filter based methods^18^, crucial for maintaining the benefits of increased dimensions in the state space. In the case where filters are used to distinguish a single basis element from all others, the probability to choose the filter that corresponds to the sent signal state is given by 2/*d*. As such, using a filter based detection, the sifting loss grows with the dimension *d*, unlike with a deterministic detector which would be unaffected by the larger state space. For example, in the demonstration shown in Fig. 4, filtering one mode at the time would only yield a raw key that is, on average, 50 bits long; that is because a maximum of two vector modes can be distinguished in a single shot measurement using a *q*-plate as a filter^16^.

## Discussion

In this work we presented a deterministic measurement scheme where many and single photon local Bell states in the form of vector vortex modes were sorted and detected with, in principle, unit detection probability. Since each mode maps to a unique position in the spatial domain, such a detection will not suffer from any dimension-dependent loss. In QKD, where photon loss as a result of the measurement procedure affects the efficiency of the transmission, our scheme makes it possible to increase the dimensionality without compromising on the sifting rate. We point out that our scheme would likewise increase the signal-to-noise of classical mode-division multiplexing communication systems: rather than distribute the signal across *d* modes, each with 1/*d* of the signal, we can achieve full signal on each mode with a factor *d* greater signal-to-noise ratio^35^. A major advantage we offer is that all modes in our Hilbert space are eigenmodes of free-space^36,37^ and optical fibre^22^, thus facilitating long distance applications outside the laboratory environment.

## Methods

### Security analysis

From the measured crosstalk matrices in Fig. 3(c,d), we performed a security analysis on our QKD scheme in dimensions *d* = 4 for the two OAM subspaces (±1 and ±10). The results of the analysis are summarised in Table 2. From the measured detection fidelity *F*, we computed the mutual information between Alice and Bob in *d*-dimensions as follows^19^

19 $${I}_{AB}={\mathrm{log}}_{2}\,(d)+F\,{\mathrm{log}}_{2}\,(F)+\mathrm{(1}-F)\,{\mathrm{log}}_{2}\,(\frac{1-F}{d-1}).$$

The measured *I*_*AB*_ for *d* = 4 is nearly double (1.7×) that of the maximum achievable with only qubit states (1). Assuming a third party, Eve, uses an ideal quantum cloning machine to extract information, the associated cloning fidelity, *F*_*E*_, in *d*-dimensions is given by^19^

20 $${F}_{E}=\frac{F}{d}+\frac{(d-\mathrm{1)(1}-F)}{d}+\frac{2\sqrt{(d-\mathrm{1)}\,F\mathrm{(1}-F)}}{d}.$$

With increasing dimensions, the four dimensional protocol reduces the efficiency of Eve’s cloning machine to as low as 0.38 well below the maximum limit in a two-dimensional protocol (0.5) Thus, increasing the dimensionality of QKD protocols does indeed have, in addition to higher mutual information capacity, higher robustness to cloning based attacks. We emphasise here that we take the case of cloning based attacks was used to illustrate a proof-of-concept; that is, increasing the dimensionality of quantum protocols provides added security benefits.

The mutual information shared between Alice and Bob, conditioned on Bob’s error–that is, Bob making a wrong measurement is as a result of Eve extracting the correct information–is computed in *d*-dimension as follows^10^

21 $${I}_{AE}={\mathrm{log}}_{2}\,(d)+(F+{F}_{E}-\mathrm{1)}\,{\mathrm{log}}_{2}\,(\frac{F+{F}_{E}-1}{F})+\mathrm{(1}-{F}_{E})\,{\mathrm{log}}_{2}\,(\frac{1-{F}_{E}}{(d-\mathrm{1)}F}).$$

The consequent measured quantum error rate of *Q* = 1 − *F* = 0.04 is well below the 0.11 and 0.18 bounds for unconditional security against coherent attacks in two and four dimensions^10^, respectively. The lower bound on the secret key rate, given by^38^

22 $$R={\mathrm{log}}_{2}\,(d)+2F\,{\mathrm{log}}_{2}\,(F)+\mathrm{2(1}-F)\,{\mathrm{log}}_{2}\,(\frac{1-F}{d-1}),$$

yields a value as high as 1.52 bits per photon, well above the Shannon limit of one bit per photon achievable with qubit states. While the security of the protocol can be increased with privacy amplification, the measured four-dimensional secret key rate demonstrates the potential of such entangled modes for high bandwidth quantum communication.

### Data availability statement

All data regarding the work presented here are available upon request to the corresponding author.
